# Detection of MYD88 and CXCR4 mutations in cell-free DNA of patients with IgM monoclonal gammopathies

**DOI:** 10.1038/s41375-018-0197-7

**Published:** 2018-07-19

**Authors:** Tina Bagratuni, Ioannis Ntanasis-Stathopoulos, Maria Gavriatopoulou, Nefeli Mavrianou-Koutsoukou, Christine Liacos, Dimitrios Patseas, Nikolaos Kanellias, Magdalini Migkou, Dimitrios C. Ziogas, Evangelos Eleutherakis-Papaiakovou, Maria Roussou, Despina Fotiou, Evangelos Terpos, Efstathios Kastritis, Meletios A. Dimopoulos

**Affiliations:** Department of Clinical Therapeutics, School of Medicine, National and Kapodistrian University of Athens, Athens, Greece

## Abstract

Liquid biopsyis being integrated into cancer diagnostics with profound therapeutic implications. However, its role in Waldenström’s Macroglobulinemia (WM) and IgM monoclonal gammopathies is still unclear. In this study, we evaluated the role of peripheral blood (PB) cell-free DNA (cfDNA) in characterizing the mutational status of *MYD88* and *CXCR4* of patients with IgM monoclonal gammopathies. Paired bone marrow (BM) tumor DNA (tDNA) and PB cfDNA samples from 98 patients (9 MGUS, 45 with WM in remission, 44 with smoldering WM, newly diagnosed or relapsed WM) and 10 controls with non-IgM monoclonal gammopathies were analyzed. Regarding MYD88^L265P^ mutation, 76 patients had paired tDNA and cfDNA informative samples. Among patients with WM in remission, 65% harbored the MYD88^L265P^ mutation, whereas the corresponding percentage among smoldering/newly diagnosed or relapsed WM was 92%. The overall concordance rate was 94% (72/76). For CXCR4 mutations, 65 patients had paired informative tDNA and cfDNA samples. The overall concordance rate was 90% (59/65). All controls had wild-type *MYD88* and *CXCR4*. In conclusion, PB cfDNA is a useful, minimally invasive, cost-effective, and time-effective tool for the identification of the presence of *MYD88* and *CXCR4* mutations in patients with IgM monoclonal gammopathies avoiding unnecessary BM assessment.

## Introduction

Whole-genome sequencing has identified highly recurring somatic mutations in patients with Waldenström’s Macroglobulinemia (WM) and IgM Monoclonal Gammopathy of Undetermined Significance (MGUS) [1, 2]: 76–100% of WM patients and 43–87% of those with IgM MGUS harbor a single point mutation in *MYD88* gene (rs387907272), resulting in p.Leu265Pro (L265P) amino acid change [1–5]. *MYD88* is an adapter for Toll-like and interleukin-1 (IL1) receptors, and the *MYD88*^*L265P*^ mutation results in constitutive activation of nuclear factor κB through IL1 receptor-associated kinase and Bruton’s tyrosine kinase (BTK) [1, 6–8]. The presence of *MYD88*^*L265P*^ mutation provides a significant evidence for the diagnosis of WM but has also been associated with improved probability and quality of clinical responses to the BTK inhibitor ibrutinib [9–11]. Recurrent activating somatic mutations (frameshift or nonsense) are also found in C–X–C chemokine receptor type 4 (*CXCR4*) gene in a frequency of about 30–40% of patients with WM and less often in those with IgM MGUS, representing the second most mutated gene in WM [2, 3, 12]. CXCR4 is a G-protein-coupled receptor, acting as a key regulator of cell trafficking in heamatopoietic stem cells and clonal B cells, also interacting with the related ligand stromal-derived factor 1 [13–16]. Studies have shown the relevance of specific CXCR4 mutations to enhanced WM cell dissemination leading to in vivo disease progression as well as increased resistance to ibrutinib treatment [17–19]. Both *MYD88* and *CXCR4* mutations are detected in the bone marrow (BM), although there have been attempts to detect them in CD19^+^-selected cells in the peripheral blood (PB) of patients with IgM monoclonal gammopathies [20, 21].

Analysis of tumor-derived circulating nucleic acids, such as circulating cell-free tumor DNA (cfDNA) (termed also as liquid biopsy) is currently being integrated in diagnosis, prognosis, disease monitoring, and early detection of clonal evolution of several different solid tumors [22]. cfDNA may reflect the genomic alterations within the whole tumor compartment and may also substitute the need for invasive tissue sampling in specific situations [23]. In hematological malignancies, cfDNA may be used as a surrogate marker of response to treatment, minimal residual disease (MRD), and early detection of clonal evolution and relapse in patients with B-cell Non-Hodgkin Lymphoma and Chronic Lymphocytic Leukemia [24–26]. There is also accumulating evidence indicating the role of cfDNA in patients with multiple myeloma (MM) as a universal marker of tumor burden with prognostic implications [27, 28].

The aim of our study was to investigate the role of cfDNA in the characterization of the mutational status of *MYD88* and *CXCR4* of patients with IgM monoclonal gammopathies.

## Materials and methods

PB (10–12 mL) and BM aspirates (5–10 mL) were collected from 98 patients, including 24 patients with smoldering WM, 11 with symptomatic WM, 9 with IgM MGUS, as well as 10 control donors with non-IgM monoclonal gammopathies; their characteristics are summarized in Table 1. The BM infiltration in patients with smoldering WM was 30% (range 12–80%), newly diagnosed symptomatic WM was 60% (range 14–90%), and in WM in relapse was 38% (range 20–85%). PB was collected in EDTA tubes and processed immediately for DNA extraction using the MagMax cell-free DNA isolation kit (Thermo Fisher Scientific), according to manufacturer’s instructions. The quantity of cfDNA was measured by qubit fluorometer using the HS dsDNA kit (Thermo Fisher Scientific). cfDNA concentration varied between 6 and 80 ng/μL. BM aspirates were collected at the same time with PB, and were processed for CD19 enrichment [1, 29]. Briefly, mononuclear cells from BM aspirates were isolated by Ficoll-Paque gradient centrifugation followed by positive selection using CD19 magnetic beads BM (Miltenyi Biotech). DNA of CD19^+^-selected cells (tDNA) was extracted using Allprep DNA/RNA mini kit (QIAGEN, Valencia, CA) and quantified using the HS dsDNA kit. The paired tDNA and cfDNA samples from patients and controls were analyzed for the mutation detection of *MYD88*^*L265P*^ in the exon 5 of *MYD88* gene and for *CXCR4* gene in all exons.

**Table 1:** Patient characteristics

*IgM patients* (*n*)	98
IgM MGUS	9
Asymptomatic WM	23
Newly diagnosed symptomatic WM	12
WM in relapse	9
WM during Tx and in remission	17
WM post Tx	28
Kappa/Lamda	79/19
Age (median, range in years)	72.5 (23–88)
Bone marrow infiltration (%; median, range)	25 (0–90)
IgM levels (median, range in mg/dL)	1260 (85–7810)
*Controls* (*n*)	10
Symptomatic MM (*n*)	5
Smoldering MM (*n*)	2
Non-IgM MGUS (*n*)	3
Age (years; median, range)	67 (57–81)

*IgM* immunoglobulin M, *WM* Waldenström’s Macroglobulinemia, *MM* multiple myeloma, *MGUS* monoclonal gammopathy of undetermined significance, *LC* light chain

The detection of *L265P* mutation by allele-specific PCR (AS-PCR) and direct sequencing in both tDNA and cfDNA was performed as previously described [30]. Specifically PCR was performed with up to 15 ng of cfDNA or tDNA (depending on DNA availability in each case). Thermal cycling conditions were as follows: five minutes at 94 °C followed by 35 cycles of amplification using 60 s at 94 °C, 60 s at 60 °C, and 60 s at 72 °C, with subsequent 5 min extension at 72 °C. AS-PCR was performed with specific forward primers with a single base substitution at the end of the primer: MYDW-F, 5′-GTGCCCATCAGAAGCGCCT-3′ (wild type), MYDM-F, 5′-GTGCCCATCAGAAGCGCCC-3′ (mutant), and MYD-R, 5′-GACGTGTCTGTGAAGTTGGCATCTC-3′ (reverse). *MYD88* wild-type and *MYD88* mutant amplified 224 base pair (bp) products and were visualized in 1.5% EtBr agarose gel. The PCR conditions for MYD88 used for direct sequencing were the same as above. The reverse primer was the same as for the AS-PCR, while the forward sequence for sequencing was MYD_F, 5′-GGGATATGCTGAACTAAGTTGCCAC-3′ (forward). The amplification of the above PCR resulted in a 726  bp product. Sequencing of CXCR4 was performed using the following primers: CXCR4_1, 5′-GCTGAATTGGAAGTGAATGTCC-3′ (forward), CXCR4_1, 5′-GTCATCTACACAGTCAACCTCT-3′ (reverse), CXCR4_2, 5′-CCGTGGCAAACTGGTACTTT-3′ (forward), CXCR4_2, 5′-TCACTCCAGCTAACACAGATG-3′ (reverse). Amplicon sizes for CXCR4 1 and 2 were 389 and 775 bp, respectively. As cfDNA size is small (~166  bp), we designed primers for CXCR4 gene targeting smaller amplicon sizes (100–220  bp) and amplified cfDNA samples that were largely degraded. The primers used are shown in Table 2.

**Table 2:** CXCR4 primers

Name	Primer	Direction	Amplicon size (bp)
CXCR4_1	5′-ACTGGGTTAATGCTTGCTGA-3′	F	184
	5′-TGCCCACCATCTACTCCATC-3′	R	
CXCR4_2	5′- GCCCACCATCTACTCCATCA-3′	F	158
	5′-TGTCATCACGCTTCCCTTCT-3′	R	
CXCR4_3	5′-TGTCATCACGCTTCCCTTCT-3′	F	219
	5′-AAGGTGGTCTATGTTGGCG-3′	R	
CXCR4_4	5′-AAGGTGGTCTATGTTGGCGT-3′	F	162
	5′-TTCAGCACATCATGGTTGGC-3′	R	
CXCR4_5	5′-ATCTGTGACCGCTTCTACCC-3′	F	216
	5′-CCTGTTGGCTGCCTTACTAC-3′	R	
CXCR4_6	5′-CTCATCCTGGCTTTCTTCGC-3′	F	173
	5′-TGTTGTCTGAACCCCATCCT-3′	R	
CXCR4_7	5′-CATCCTCTATGCTTTCCTTGGAG-3′	F	171
	5′-TCACTCCAGCTAACACAGATG-3′	R	

The thermal conditions for the above reactions were the same as in MYD88. Amplification and sequencing of MYD88 and CXCR4 for each sample was performed once, unless unsuccessful result required the repetition of either method. The study was approved by the institutional review board and all patients provided written informed consent for sample collection and analysis.

## Results

### *MYD88* genotype assessment in cfDNA and tDNA

Of the 98 patients with IgM monoclonal gammopathies, the mutational status of *MYD88* could be evaluated in 84 patients (84/98 patients, 86%) by tDNA and in 84 patients (84/98 patients, 86%) by cfDNA. In 14 patients tDNA (14/98, 14%) and in 14 patients cfDNA (14/98, 14%), *MYD88* status could not be assessed due to inability of BM aspiration or to low DNA quantity, respectively. Most patients with insufficient tDNA had very low BM infiltration by lymphoplasmacytic cells: three patients had IgM MGUS, four had smoldering WM (sWM), one was newly diagnosed WM, and six had WM in remission after therapy; however, in 57% of these patients (8/14) cfDNA was informative. Among the 14 patients in which cfDNA could not be obtained, 2 had IgM MGUS, 3 were sWM, 1 was newly diagnosed (ND)WM with relatively low BM infiltration (~12%), and 8 had WM in remission.

**Fig. 1:**
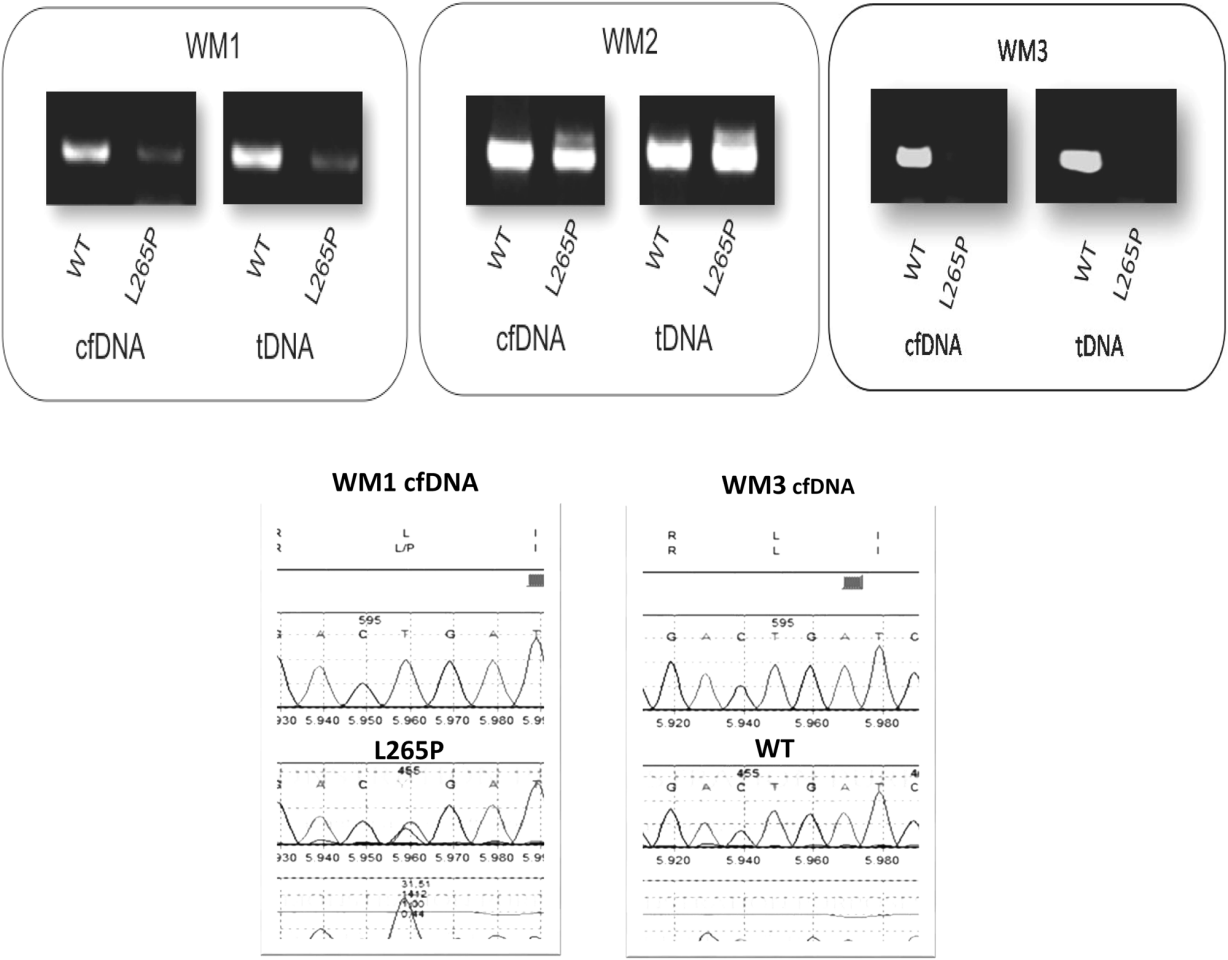
AS-PCR and Sanger sequencing for MYD88 L265P in tDNA and cfDNA. WM1 and WM2 present in both wild-type and mutant products, while WM displays oonly the wild-type allele. WM1 and WM3 cfDNA sequencing analysis is also displayed

We next sought to clarify the feasibility of using cfDNA for the detection of *MYD88*^*L265P*^ mutation in 84 patients where cfDNA was available. The mutation was detected in the cfDNA of 68 out 84 (81%) patients. As shown in Fig. 1, the somatic mutation L265P in the *MYD88* gene is clearly detectable in cfDNA samples by AS-PCR agarose gel electrophoresis. To verify the validity of the results seen by AS-PCR in the agarose gel electrophoresis, the mutated product was also analyzed by direct sequencing. The *MYD88* mutational analysis was also confirmed by direct sequencing, where the quality of the images is also shown in Fig. 1. The feasibility of using cfDNA for determining the *MYD88* mutation status is equally informative to that determined by tDNA (Fig. 1). In samples with MYD88^WT^, only the wild-type amplicon product was observed (Fig. 1).

**Fig. 2:**
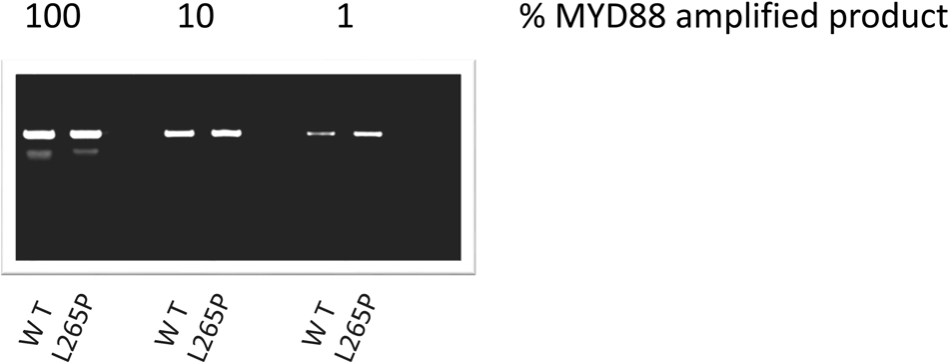
The sensitivity assessment of AS-PCR in cfDNA *MYD88* wild-type and mutated product as seen on a 1.5% agarose-gel electrophoresis. Both the wild-type and the mutant alleles were detected to a dilution of 1%

To determine the sensitivity of the AS-PCR assay for the cfDNA samples we performed serial dilutions of a mutated cfDNA product. The sensitivity assessment of AS-PCR in cfDNA demonstrated that *MYD88*^*L265P*^ mutation was detectable in sample product with a low DNA concentration (5 ng) in a dilution of up to 1% (as low as 0.05 ng of DNA), where the mutated band is clearly detectable (Fig. 2). Our results show that further dilutions are possible. In parallel we examined the use of both conventional AS-PCR and direct sequencing for the detection of the *MYD88*^*L265P*^ mutation in tDNA derived from CD19-selected BM cells. The mutation was detected in 71 out of 84 patients (84%). All patients in both cfDNA and tDNA, positive or negative for L265P MYD88 by AS-PCR, were also positive or negative by direct sequencing, respectively. The results showed complete reproducibility among replicates between different runs.

Among tested patients, 76 patients had both tDNA and cfDNA informative samples. *MYD88*^*L265P*^ mutation was detected in 62 out of 76 patients (82%) in both tDNA and cfDNA; in 4 out of 76 (5%) the mutation was seen in tDNA but not in cfDNA and 10 out of 76 (13%) patients harbored the *MYD88*^*WT*^ genotype both in tDNA and cfDNA. Thus, the overall concordance between tDNA and cfDNA for *MYD88* genotype was 94% (72 out of 76 patients). Among patients with IgM MGUS, WM in remission, and sWM/NDWM/relapsed (RR) WM, the concordance rates were 100% (6 out of 6 patients), 97% (33 out 34 patients), and 91% (33 out of 36 patients), respectively. The frequency of detection of *MYD88*^*L265P*^ in tDNA was very similar for patients with sWM (95% *MYD88*^*L265P*^) vs. NDWM (91%) vs. WM in relapse (100% *MYD88*^*L265P*^) (*p* = 0.653). Regarding detection in cfDNA was also similar for patients with sWM (82% *MYD88*^*L265P*^) vs. NDWM (73%) vs. WM in relapse (100% *MYD88*^*L265P*^) (*p* = 0.439). Overall, the *MYD88*^*L265P*^ mutation by either cfDNA or tDNA was present in 6 out of 6 MGUS patients (100%), 27 out of 34 patients with disease in remission (79%), and in 36 out of 44 patients with smoldering or newly diagnosed or relapsed disease (81%).

Regarding the control group of non-IgM MGUS and MM patients, there were no *MYD88* mutations detected in tDNA (8 out of 8 for *MYD88* and 8 out of 8 patients for *CXCR4*) and cfDNA (6 out of 6 patients for *MYD88* and 7 out of 7 patients for *CXCR4*). One patient with marginal zone lymphoma harbored the *MYD88*^*L265P*^ mutation detected both in tDNA and cfDNA.

We also evaluated whether the degree of BM infiltration, which grossly reflects the burden of the clone and IgM levels were associated with mutation detection rates. In univariate analysis (*t*-test, two-sided), the probability of identifying MYD88^L265P^ in CD19^+^ cells was marginally associated with the BM infiltration (*p* = 0.062) and was associated with IgM levels (*p* = 0.012). Regarding MYD88^L265P^ detection by cfDNA this was not significantly associated with BM infiltration (*p* = 0.126) and was associated with IgM levels (*p* = 0.045). The amount of cfDNA was marginally associated with BM infiltration (*p* = 0.083) and not with IgM levels (*p* = 0.188); on the contrary, and as expected, there was an association of collected CD19^+^ cells with BM infiltration (*p* = 0.012), but not with IgM levels (*p* = 0.074). These results further indicate the potential usefulness of cfDNA even in patients with low levels of BM infiltration.

### *CXCR4* genotype assessment in cfDNA and tDNA

**Fig. 3:**
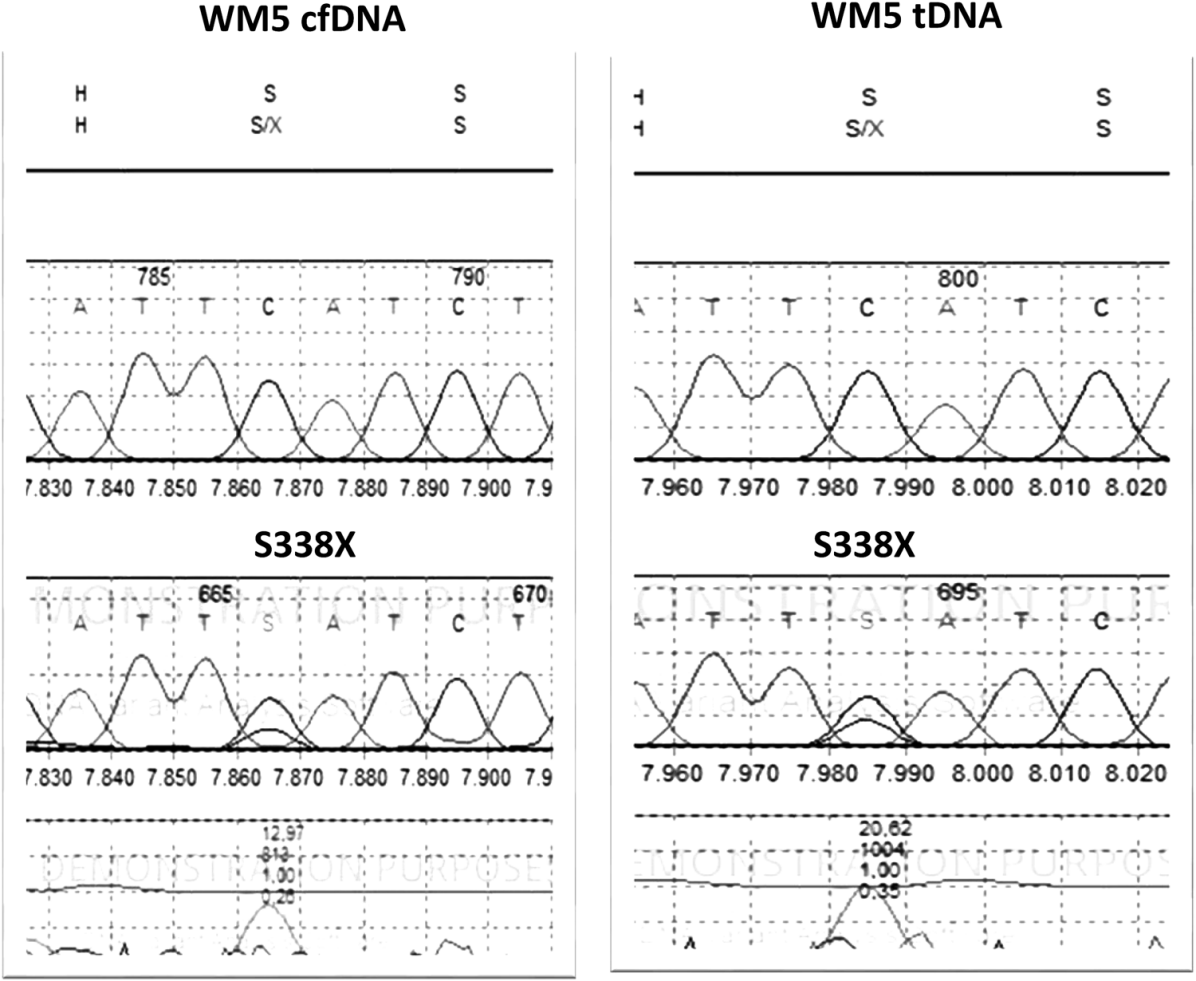
Sanger sequencing of cfDNA and tDNA for mutations in CXCR4. WM5 is characterized by the pathogenic S338X mutation as displayed in both tDNA and cfDNA by the sequencing chromatogram

We next examined the feasibility of using cfDNA in detecting mutations in *CXCR4* gene. Of the 98 patients with IgM monoclonal gammopathies, mutational screening of *CXCR4* gene could be evaluated in 79 patients with cfDNA (79/98 patients, 80%) and in 73 patients with tDNA (73/98 patients, 74%). The assessment of *CXCR4* mutations in both tDNA and cfDNA was feasible in 65 patients (65 out of 98 patients, 66%). In eleven patients (11 out of 65, 17%), mutations were detected in both paired samples. *CXCR4* mutations by either cfDNA or tDNA were present in 2 out of 9 MGUS (22%) patients, 10 out of 37 (27%) patients with disease in remission, and in 6 out of 29 (20%) patients with sWM/NDWM/RRWM. The pathogenic mutation *S338X* (rs104893626) was present in three patients in one patient with sWM and in two with NDWM. Figure 3 illustrates the sequencing chromatogram of S338X mutation as presented in tDNA and cfDNA. The S338X mutation results from a single nucleotide C to G change leading to a predicted stop codon in place of a serine at amino acid position 338. Mutations found in CXCR4 gene are presented in Table 3. Synonymous mutations were found in five patients. Five patients harbored more than one mutation. Fifty-six patients (56 out of 65 patients) were characterized as *CXCR4*^*WT*^ based on both tDNA and cfDNA sequencing. In 3 out of 65 patients, the *E343D*, *H228Q*, and *L50X* truncating mutations were detected in tDNA but not in cfDNA. The *L210P* mutation (1 out of 65 patients) and the V54G, K110R, and V99G mutations (1 out of 65 patients) were detected in the cfDNA but not in tDNA. Overall, the concordance rate between tDNA and cfDNA was 90% (59 out of 65 patients). The concordance rate was 100% among patients with IgM MGUS (7 out of 7 patients), 82% among WM patients in remission (23 out of 28 patients), and 93% among patients with sWM/NDWM/RRWM (28 out of 30 patients). In the four patients with informative tDNA only, one patient presented many polymorphisms in *CXCR4* gene, while among eight patients with uninformative tDNA evaluation of cfDNA revealed the *V99G* mutation (1 out of 65 patients) and V82G (1 out of 65 patients). All but S338X (3/65 patients) and V99G (2/65 patients) polymorphisms were presented in one patient. Table 4 illustrates the information regarding our reported polymorphisms in CXCR4 gene. Of these mutations, only S338X and E343D are located in the C-terminal portion, while the rest affect other portions of the protein. As the C-terminal portion plays a critical role in the regulation of signal transduction and CXCR4 expression [31], further analysis is under investigation to determine whether these mutations are tumor specific and clinically relevant to WM pathogenesis. Further analysis will also be conducted for the rest of the detected mutations, especially those which seem to have a higher functional impact according to Provean data (Table 4).

**Table 3:** CXCR4 mutation predictions

Mutation (Hg19)	Mutation (Hg38)	Gene	Provean number	Prediction	Reported SNP
P27T	P31T	CXCR4	−2.986	Deleterious	Not reported
F29L	F33L	CXCR4	−0.778	Neutral	Not reported
L50X	L54X	CXCR4		Pathogenic	Not reported
I53L	I57L	CXCR4	−0.121	Neutral	rs56400844
V54G	V58G	CXCR4	−4.11	Deleterious	Not reported
V82G	V86G	CXCR4	−5.5	Deleterious	Not reported
V99G	V103G	CXCR4	−1.952	Neutral	Not reported
K110R	K114R	CXCR4	−2.4	Neutral	Not reported
L136Q	L140Q	CXCR4	−5.5	Deleterious	Not reported
H228Q	H232Q	CXCR4	−0.306	Neutral	rs762625278
S338X	S342X	CXCR4		Pathogenic	rs104893626
E343D	E347D	CXCR4	−1.244	Neutral	Not reported

**Table 4:** Rates of detection of *MYD88*^L265P^ and of mutations in CXCR4 in CD19^+^-selected cells and in cfDNA, according to different disease states in 98 patients with monoclonal IgM

	CD19+ MYD88L65P	cfDNA MYD88L265P	CD19+ CXCR4mut	cfDNA CXCR4mut
IgM MGUS	(*N* = 6) 100%	(*N* = 7) 86%	(*N* = 7) 29%	(*N* = 9) 22%
sWM	(*N* = 21) 95%	(*N* = 22) 82%	(*N* = 17) 29%	(*N* = 16) 19%
NDsWM	(*N* = 11) 91%	(*N* = 11) 73%	(*N* = 8) 38%	(*N* = 12) 25%
WM in relapse	(*N* = 9) 100%	(*N* = 9) 100%	(*N* = 8) 0%	(*N* = 9) 0%
WM during active therapy	(*N* = 13) 69%	(*N* = 13) 62%	(*N* = 11) 27%	(*N* = 15) 27%
WM post therapy	(*N* = 26) 81%	(*N* = 24) 83%	(*N* = 22) 18%	(*N* = 20) 10%
Overall	(*N* = 86) 87%	(*N* = 86) 80%	(*N* = 73) 23%	(*N* = 81) 17%

A number of samples with sufficient cfDNA were also tested with primers targeting smaller gene amplicons. This was due to the fact that cfDNA size is relatively small and potentially degraded, and hence may not able to support larger amplicon size. The smaller amplicon targeting primers were tested in 4 samples which had sufficient cfDNA and in which sequencing had been unsuccessful, and in 10 samples which had been successfully sequenced (5 cfDNA and 5 tDNA) in order to test concordance with our first set of primers targeting larger amplicons. We found that samples in which sequencing was not successful with the first set of primers, did not also work with the primers targeting small amplicons. Sequencing with the small amplicon targeting primers gave the same results with samples amplified with the first set of primers.

Regarding the control group of non-IgM MGUS and MM patients, no *CXCR4* mutations were detected in tDNA (8 out of 8 patients for *CXCR4*) and cfDNA (7 out of 7 patients for *CXCR4*).

## Discussion

This is the first study to evaluate the feasibility of detecting somatic mutations of *MYD88* and *CXCR4* in the cfDNA derived from the PB plasma of patients with IgM monoclonal gammopathies. The evaluation of cfDNA for mutational characterization and monitoring of disease burden in other hematologic malignancies such as MM has been recently described and shown that cfDNA levels are significantly higher in MM patients compared to other type of cancers [27, 28, 32]. In addition it has been shown that the sensitivity of detecting mutations in plasma was significantly higher compared to the detection in the BM [32]. Although BM examination is required for the diagnosis of WM, the application of cfDNA could help in monitoring the response to therapy as well as MRD, avoiding the repeated BM sampling. Further studies with larger cohort of WM patients will be performed, to evaluate if this is also the case for detecting mutations in the plasma of these patients.

In our study, we found that in patients with IgM monoclonal gammopathies the MYD88^L265P^ mutation and mutations in *CXCR4* gene in cfDNA can be detected with a high concordance of 94 and 90%, respectively, compared to those detected in the tDNA. These high concordance rates indicate that cfDNA testing provides are liable diagnostic tool which could replace BM testing in many cases. Table 4 illustrates the rates of detection of *MYD88*^L265P^ and of mutations in CXCR4 in CD19^+^-selected cells and in cfDNA according to different disease states. Additional studies are ongoing, to further determine whether these results may improve with further optimizations and more sensitive approaches. In addition collection of serial samples from the same patient before, during, and after treatment is ongoing, which would provide significant information regarding the use of cfDNA as a tool for molecular monitoring, and determine whether in patients with MYD88^L265P^ could be used for sensitive detection of MRD after obtaining a deep response.

The prevalence of MYD88^L265P^ (82%) in our cohort is comparable to the rates observed in other studies [5, 29, 33, 34]. The prevalence rates are also comparable to those achieved using CD19-selected cells from PB [20, 21]. Although previous studies using unselected PB cells from untreated WM patients have shown that the MYD88 L265P mutation can be detected, the detection sensitivity was so low (39.5%) that they were prompted to examine the mutation in CD19-selected PB cells using magnetic bead isolation for AS-PCR [20]. In regard to the detection sensitivity of MYD88 L265P mutation, it has been previously reported that although lower BM burden (<10% infiltrate) in patients could result to false negative results, AS-PCR is sensitive enough to detect the L265P mutation in tumor cells as low as 1.25% [35]. Our results confirm these findings, since in patients with BM infiltrate as low as 5%, the L265P mutation could be detected in PB cfDNA by both AS-PCR and direct sequencing.

This minimally invasive method could be considered as a cost-effective approach, avoiding the discomfort and risks associated with BM aspiration and the time-consuming tDNA acquirement through CD19 processing (from BM or PB). Most importantly, cfDNA seems to be representative of both the extramedullary disease and the whole BM compartment and not just of a specific site of sampling which might inadequately reflect clonal heterogeneity and inconsistent distribution of clonal cells within the BM, which is typically the case during a BM aspiration or biopsy. The application of cfDNA testing demonstrates the potential for a minimally invasive platform with an increasing range of application in stratifying patients according to their genetic identity. Thus, PB cfDNA could become a useful tool for the initial assessment of *MYD88*^*L265P*^ and *CXCR4* mutations by reflecting the overall tumor burden in patients with WM. Furthermore, cfDNA could prevent a repeat BM examination in patients diagnosed with IgM monoclonal gammopathies and unknown mutational status by minimizing the conventional BM-derived tDNA mutation detection to those patients with indeterminate results. To confirm our hypothesis, a larger cohort of patients is tested, in order to obtain a more comprehensive genetic profiling of cfDNA and tDNA in serial samples and assess tumor dynamics by means of cfDNA.

In conclusion, the use of PB cfDNA provides a minimally invasive, but informative and sensitive tool for the assessment of mutational status of MYD88 and CXCR4 of patients with IgM monoclonal gammopathies. Importantly, cfDNA results show high concordance with the results obtained by assessment of BM-derived CD19-positive cells and can be used for the assessment of patients with IgM monoclonal gammopathies.

## Key Points

- *MYD88* and *CXCR4* mutations were detected in cfDNA of patients with IgM monoclonal gammopathies in high concordance to bone marrow tumor DNA.
- cfDNA is a minimally invasive method for assessing the *MYD88* and *CXCR4* mutational status that may reduce the need for bone marrow aspiration.
